# Survey Email Scheduling and Monitoring in eRCTs (SESAMe): A Digital Tool to Improve Data Collection in Randomized Controlled Clinical Trials

**DOI:** 10.2196/jmir.6560

**Published:** 2016-11-22

**Authors:** Trygve Skonnord, Finn Steen, Holgeir Skjeie, Arne Fetveit, Mette Brekke, Atle Klovning

**Affiliations:** ^1^ Institute of Health and Society Department of General Practice University of Oslo Oslo Norway; ^2^ Centre for Medical Web Research Oslo Norway; ^3^ Centre for Medical Web Research Hong Kong China

**Keywords:** randomized controlled trials, data collection, surveys and questionnaires, quality improvement, sample size, Internet, email, text messaging

## Abstract

**Background:**

Electronic questionnaires can ease data collection in randomized controlled trials (RCTs) in clinical practice. We found no existing software that could automate the sending of emails to participants enrolled into an RCT at different study participant inclusion time points.

**Objective:**

Our aim was to develop suitable software to facilitate data collection in an ongoing multicenter RCT of low back pain (the Acuback study). For the Acuback study, we determined that we would need to send a total of 5130 emails to 270 patients recruited at different centers and at 19 different time points.

**Methods:**

The first version of the software was tested in a pilot study in November 2013 but was unable to deliver multiuser or Web-based access. We resolved these shortcomings in the next version, which we tested on the Web in February 2014. Our new version was able to schedule and send the required emails in the full-scale Acuback trial that started in March 2014. The system architecture evolved through an iterative, inductive process between the project study leader and the software programmer. The program was tested and updated when errors occurred. To evaluate the development of the software, we used a logbook, a research assistant dialogue, and Acuback trial participant queries.

**Results:**

We have developed a Web-based app, Survey Email Scheduling and Monitoring in eRCTs (SESAMe), that monitors responses in electronic surveys and sends reminders by emails or text messages (short message service, SMS) to participants. The overall response rate for the 19 surveys in the Acuback study increased from 76.4% (655/857) before we introduced reminders to 93.11% (1149/1234) after the new function (*P*<.001). Further development will aim at securing encryption and data storage.

**Conclusions:**

The SESAMe software facilitates consecutive patient data collection in RCTs and can be used to increase response rates and quality of research, both in general practice and in other clinical trial settings.

## Introduction

A common problem for clinical research in general practice is the ability to conduct randomized controlled trials (RCTs) with large enough sample sizes [1]. Interventions usually take place in small and busy practices, and researchers often need to organize their study by themselves [1-3]. Funding is often insufficient for employing research assistants who can conduct telephone interviews and send out reminders, unless the research is organized in a dedicated network [4,5]. A high degree of response in a trial is essential to keep the sample size sufficient, and a substantial level of nonresponse may lead to bias and less accurate data [6]. Such factors are limiting the quality, number, and progression of conducting RCTs in general practice [7]. Electronic surveys (e-surveys) greatly facilitate data collection by combining survey research and modern technology [8]. A major advantage of the use of e-surveys in research is their potential to increase the amount of data that can be collected at a low cost [9]. However, a disadvantage is that it can be challenging to secure a high response rate [9]. Jansen et al stated that questionnaires based on emails are effective methods to acquire time-specific responses, even if the compliance at specific time points might be affected [10].

Documentation of how new digital tools for clinical trials have been developed is scarce [11,12], but recent evaluations of some digital tools are available [13-15]. During the 2nd Clinical Trials Methodology Conference in 2013, McPherson et al discussed whether to use a commercial system or build one’s own software for use in clinical trials [16]. Keding et al [17] examined the effectiveness of short message service (SMS) [18] reminders on patient response rates, and rather surprisingly concluded that such reminders did not improve the response rates substantially. In a Cochrane review from 2009, Edwards et al explored different ways to increase response rates in postal and electronic questionnaires [6]. They identified 32 trials with 27 different strategies to increase response in electronic questionnaires, but none of them was about reminders. However, for postal surveys with SMS reminders, the odds of response increased by half compared with a postcard reminder.

When planning a multicenter RCT carried out in general practice [19], we struggled to find existing software that could help automate the email distribution of survey forms. We searched for, and tested, several software apps enabling survey deployment by using email software. Some of the apps were free (shareware), while others could be purchased or needed a subscription. However, all the software we tested required either that every participant had to receive the same email at the same time, or that each email had to be set up individually.

The power calculation for our study determined that we needed to include 270 patients consecutively. To collect data by electronic questionnaires at 19 specific time points within a predefined period before treatment and at a 1-year follow-up, we would have needed to send out 5130 separate emails for all questionnaires—a process that necessarily had to be automated. In the absence of adequate programs that were able to do this, we decided to develop our own software.

We aimed to develop software that would automate sending of emails with links to e-surveys, thus improving the quality of data collection and increasing the response rate to secure sufficient statistical power. This paper describes the results.

## Methods

The first version of the software was tested in a pilot study in November 2013. We developed 2 software components: an Excel (Microsoft Corporation) spreadsheet written in Microsoft Visual Basic and a server component based on Red Hat (a server operating system; Red Hat, Inc), PHP (a programming language), and MySQL (a database; Oracle Corporation). The connectivity between the user interface (the Excel spreadsheet) and the server was achieved using Open Database Connectivity. This required that the program needed to be downloaded and run from a designated laptop computer with Internet access. Every time a participant was included, the project leader (TS) had to log on to the computer and open the program to initiate the sending of emails. The software could schedule the sending of the emails with links to the surveys made in the open source program LimeSurvey (LimeSurvey GmbH) and was called Survey Email Scheduler or SES. However, in addition to the vulnerability discussed above, the software was unable to deliver multiuser and Web-based access, thereby limiting its use in larger RCTs. We solved this in the next version of the program, which we tested on the Web in February 2014. It now schedules and sends the required emails in the full-scale Acuback trial that started in March 2014 (trial registration NCT01439412) [19].

The system architecture evolved through an iterative, inductive process between the project leader (TS) and the software programmer (FS). The researcher defined the premises and the software needs for sending out multiple emails at predefined time points, and the programmer offered solutions based on the technical possibilities. The researcher was naive to programming and the programmer was research naive. Through this mutual process, they uncovered the limitations both in practical research and in programming. The program was tested, improved, and retested. This process was iterated throughout the main study, made possible by using different versions of the program, one on a development server and another on a production server.

**Figure 1:**
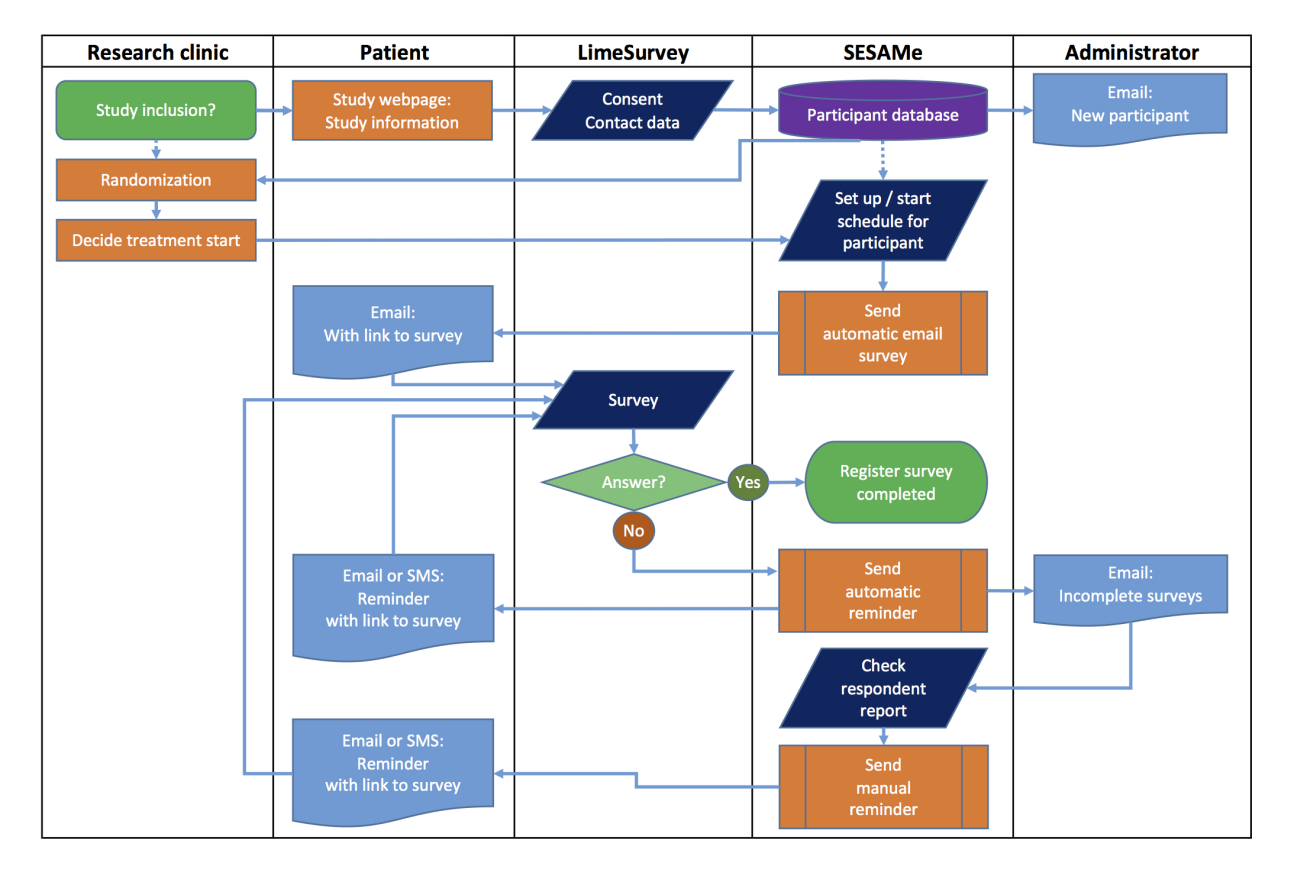
Flow chart showing the information flow during data collection in the *Survey Email Scheduling and Monitoring in eRCTs* (SESAMe) software app. SMS: short message service.

The software is now able to send reminders by either email or SMS. Reminders can be sent automatically at a given time point after the expected completion of the survey or manually through the respondent report. The project leader receives a report with the number of uncompleted surveys sent the previous day. With this improved function, all data collection and monitoring have become Internet based. We suggest signifying this type of data collection and monitoring in RCTs as an electronic randomized controlled trial (eRCT). Consequently, we also renamed the software app *Survey Email Scheduling and Monitoring in eRCTs* (SESAMe). Figure 1 illustrates the information flow during data collection, including automatic and manual reminders.

To evaluate the development of the software, we used several information sources, such as a logbook, for specific encounters and problems. We asked the research assistants using the software in the inclusion process about their experiences. We queried the participants in the Acuback trial on day 28 about how they experienced the emails and questionnaires. The continual and repeated evaluation by the software users on different levels has led to constant improvements, so we define the software development as an iterative process [20].

## Results

### The Software App

We developed a Web-based software app that schedules and sends automated emails with links to e-surveys in LimeSurvey, an open source program used by many colleges and universities worldwide. Our system is able to set a schedule either manually or by use of a template set up for the specific study.

Even if electronic questionnaires have advantages in data collection, missing data and dropouts are still a challenge. We noticed a problem with emails being registered as spam, as the participants had problems in finding them, and in using the links to the surveys. The first 11 participants in the Acuback trial received an extra questionnaire, asking whether they had experienced this problem, which email program they were using, and whether the problems had been solved. In total, 8 participants answered, and 4 of them had received 1 or more of the emails in their spam folder. We made some changes that decreased the spam grade from 2.7 to 0.0, where 5.0 is the highest possible grade [21].

### Monitoring and Reminding

**Figure 2:**
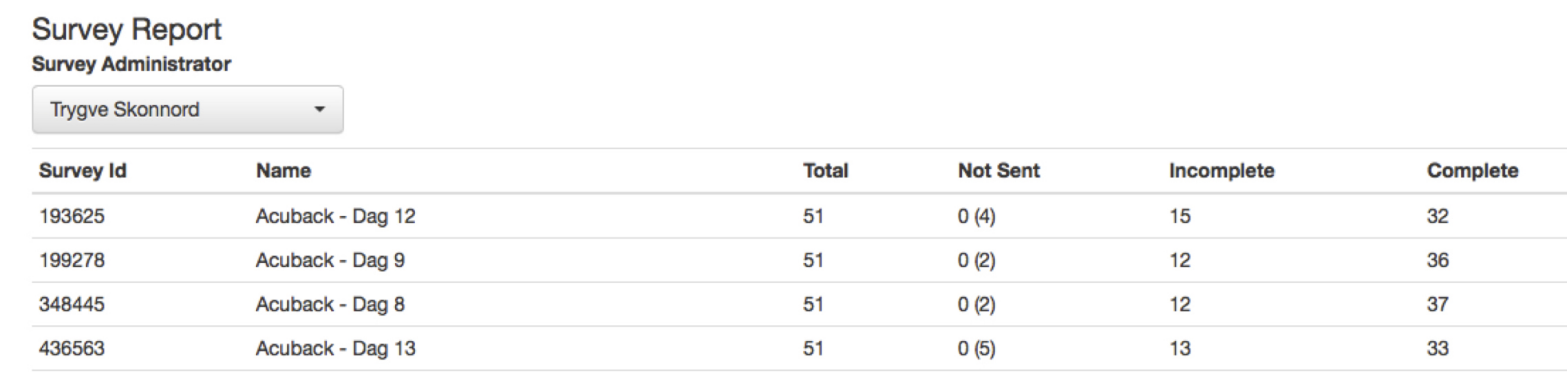
Screen dump of the survey report showing the number of completed and uncompleted surveys.

To improve the response rate, we also developed a study monitoring function for detecting missing responses. The survey report (Figure 2) shows exactly how many completed and uncompleted questionnaires have been sent from the system.

**Figure 3:**
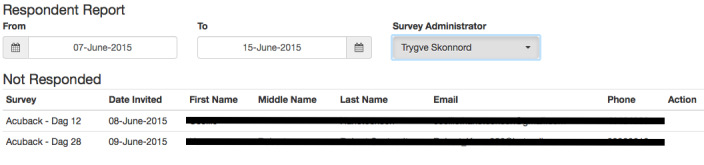
Screen dump of the respondent report showing participants who have not responded to a survey during a specified time period.

A respondent report (Figure 3) shows who has not answered the survey in a given period. The report can be extended for an individual respondent, giving data for when emails and SMSs were sent both for this specific survey and for all the surveys the participant has received.

### Increased Response Rate

The survey report in SESAMe contains information about how many surveys have been distributed, how many have not yet been sent out, and how many have been completed or not completed. We used this to compare the response rate for the surveys before and after we introduced the possibilities to send out manual or automatic reminders by email or SMS (October 11, 2014). We included 51 participants before this date and 66 participants from that date to January 21, 2016, giving a total of 117 participants. Of these, 57 (48.7%) were men and 43 (36.87%) had an education >13 years. Mean age for the participants was 43 years for men and 37 years for women.

**Figure 4:**
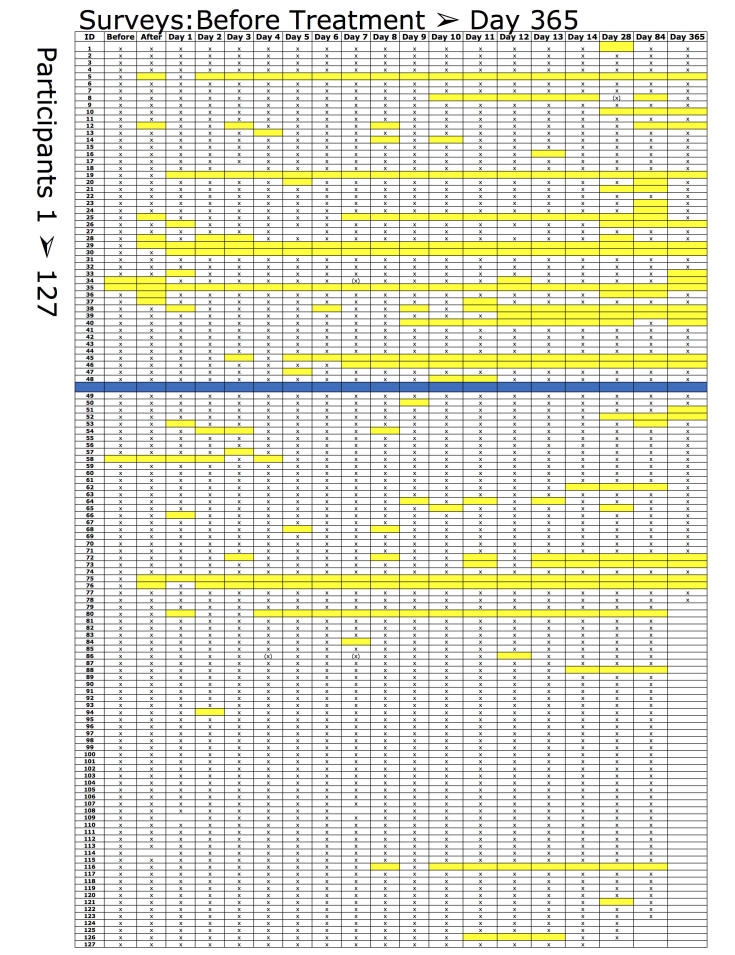
Missing answers, marked with yellow, before and after monitoring function (blue line).

With 18 surveys in the first period (no participant reached day 365), 857 emails were sent and 655 surveys were answered: a response rate of 76.4%. For the second period, 1149 of 1234 surveys were answered: a response rate of 93.11% (*P*<.001 between periods by chi square test). Figure 4 shows the increased response rate, with fewer missing answers after the software had been upgraded (blue line).

### Preferences

On day 28 (survey number 17), we posted 4 extra questions to the participants about their experiences with the questionnaires. By November 10, 2015, a total of 69 of 96 (72%) had submitted their answers to the questions. Table 1 summarizes the results. Most were satisfied with the questionnaires. When asked whether anything did not function as it should have, 9 of the 69 respondents replied “yes.” Their comments taught us that some links to the questionnaires did not function in the beginning, and 3 of them said that the emails had defaulted into their email spam folder. Most participants preferred electronic questionnaires, but this is a selected group, as they had already agreed to participate in an e-survey. We also found that participants used several kinds of devices to answer the questionnaires.

**Table 1:** The participants’ experiences and preferences with electronic questionnaires (n=69).

Questions and responses		n
**In total, how satisfied were you with the functionality of these questionnaires?**		
	Very satisfied	15
	Satisfied	29
	Neither satisfied nor dissatisfied	23
	Somewhat dissatisfied	2
	Very dissatisfied	0
**Was there anything about the emails or questionnaires that did not function as it should?**		
	No	60
	Yes	9
**Did you experience that it was fine to use electronic questionnaires, or would you have preferred to use paper questionnaires?**		
	I prefer electronic questionnaires	62
	It doesn’t matter	7
	I prefer paper questionnaires	0
**What kind of electronic devices did you use to answer the surveys?**		
	Desktop computer	17
	Portable computer	35
	Tablet	20
	Smartphone	29
	Other (smart TV)	1

### Experiences From the Users of SESAMe

In the Acuback trial, research assistants at the general practice clinics enrolled and randomly allocated the participants, and then used SESAMe to deploy emails. Finally, they answered a survey to ensure that they had completed the inclusion and reported any eventual problems with the program. For the first 111 completed surveys, only 14 assistants reported problems with randomization or email processing: 4 of them did not report the nature of the problem and 2 generalized it to be caused by “using SESAMe.” Of the research assistants, 3 reported the patients’ problem to be email being sent to the spam folder, and 2 patients did not receive the first email at all (solved by sending an SMS manually). On one occasion, the mapping from the content survey failed, and twice a patient was doubly registered through the mapping. In addition, the server once shut down during inclusion.

## Discussion

During the planning of our RCT to be conducted in general practice, we lacked appropriate software to carry out repeated electronic data collection from patients continually enrolled over a long time. Instead of converting the data collection scheme back to being paper based, we developed the software needed to automate the scheduling and sending of the emails in our trial. Such an automation process is required when participants who are consecutively included in a trial receive emails in a specific order at different time points, especially when a large number of participants is required. Using the iterative, inductive process described above, we developed a tool that we named SESAMe, which other researchers might be able to use in facilitating their data collection. For the ongoing Acuback study, SESAMe has proved to be a highly significant improvement in the follow-up of participants. The SESAMe monitoring function automates and reduces time spent on necessary control functions for project leaders of RCTs. This is of especial importance in general practice research, but other clinical researchers may also save time and cost using this tool. We presented the project at the WONCA Europe conference in Copenhagen June 2016 (Multimedia Appendix 1).

While Edwards et al found an effect of SMS reminders for postal questionnaires [6], Keding et al reported that SMS reminders for electronic questionnaires did not improve the response rates substantially [17]. This is contrary to our findings, where the response rate increased from 76% to 93% when we introduced manual and automatic reminders by email and SMS. We chose to use both messaging systems. People with mobile phones might wish to answer an SMS at once if the survey is not too extensive, but if it does not suit them to answer right away, they might forget the SMS. On the other hand, emails can be read and marked as “unread” and might be remembered later more easily.

Automatic reminders require little work and contact with the participants by the researchers, but we wonder whether this might be negative as well. Do we lose something important by reducing the “human factor” in the trial? The real-life contact between the general practitioner or research assistant and the patient might increase the response rate. In our experience, sometimes the patients forgot to answer, did not understand the questions, or got tired of the surveys. When contacted, they continued to answer the surveys because of the contact with a person who could explain the topic. SESAMe can help to identify such dropouts and can be combined with personal follow-up, either by the study administrator or by the local health personnel, who may know the patients. Unfortunately, we have not registered the number of participants receiving personal contact. Telephone calls or repeated mailing has been shown to increase the response rate when participants don’t answer the first questionnaire [6].

When participants are excluded, or withdraw from the study, we have had to delete them from the program to prevent further emails from being sent to them. To keep a good research log and flow diagram of the included participants, these participants’ records should be marked as deleted and transferred to a trash folder, together with the cause of this categorization, rather than being completely deleted. Future versions of SESAMe will provide this function.

During the process of data collection in the Acuback trial, we have observed that the type of communication and language used can be important for the response rate. This is especially relevant during the inclusion process, in each of the questionnaires, and in the emails to the participants. The researchers should ensure that all included participants understand the content of the study. If language is a problem for the target group, surveys in different languages should be considered. E-surveys are suitable for deploying parallel questionnaires. The administration of SESAMe surveys is in English, and the SMS texts can be written in different languages in the software. The surveys and email texts are arranged in LimeSurvey and can be in different languages. SESAMe can organize different languages in a trial by administrating them as parallel studies or using parallel questionnaires within LimeSurvey.

### Limitations and Strengths

The evaluation of the development process of the SESAMe software is limited because it has been a practical programming process, not anchored in validated programming theories. The increase in response rate from 76% to 93% after introducing reminders is statistically significant, using the chi square test, but we might have introduced a methodological bias because the trial included more participants in the first period after the study start, making it more difficult to follow up manually. This could have been easier later on, when fewer participants were included per week. Concerning the question of satisfaction with electronic or paper questionnaires, we admit that there was a selection bias, as we asked participants who had already agreed to use electronic forms.

The strengths of this study include the process of practical development during the initial phase of the trial, and the iterative, inductive process between the research project leader and the software developer. Furthermore, we used the practical hands-on experiences of both the researchers and the other users of the program, including the participants in the study, as input into the development process.

### Security and Further Work

The security of the data now follows strong rules, with encryption of all data from each keystroke to the server and safe storage at a well-known and serious service provider [22]. The project follows the Norwegian Health Research Act, and ethical approval was given by the Regional Ethics Committee of South-Eastern Norway (reference 2013/611/REK sør-øst A). Logging on to SESAMe demands a secure password, and you are automatically logged out after an inactive period. Persons with different roles in the trial have different levels of access to the functions. Only the study administrator has access to the monitoring function and can send out the reminders, while the health personnel who enroll patients cannot see any data after the inclusion is completed. One weakness in the present version is that each study administrator also has access to the control of other eventual trials administered in SESAMe.

In the further development of our software, we will expand it to a multiuser version, where different trials will be conducted completely separate from each other in SESAMe. We aim to further secure encryption of data transportation and to use secure data storage. Data will be transferred to 2 different servers during data collection, with 1 server made inaccessible to the researcher to prevent data manipulation. Data will be released when the trial is finished. This will prevent manipulation of the data by the researchers, which is technologically feasible today. Our final aim is to make our software available for other clinical researchers.

### Conclusions

The SESAMe software app improves study logistics by automating and monitoring data collection. This opens doors to conducting large-scale RCTs, enabling researchers to conduct high-quality clinical trials, not only in a general practice setting, as in this project, but also in other settings.

## Appendix

Multimedia Appendix 1 Presentation of the project at the Wonca Europe Conference 15-18 June 2016, Copenhagen, Denmark.

## Abbreviations

e-survey: electronic survey
eRCT: electronic randomized controlled trial
RCT: randomized controlled trial
SESAMe: Survey Email Scheduling and Monitoring in eRCTs
SMS: short message service
