# Supplementary material for: Survey Email Scheduling and Monitoring in eRCTs (SESAMe): A Digital Tool to Improve Data Collection in Randomized Controlled Clinical Trials
Source: J Med Internet Res. 2016 Nov 22;18(11):e311. doi: 10.2196/jmir.6560 (PMC5141334; doi:10.2196/jmir.6560)
Supplement: Multimedia Appendix 1 [file jmir_v18i11e311_app1.ppt]

## Slide 1
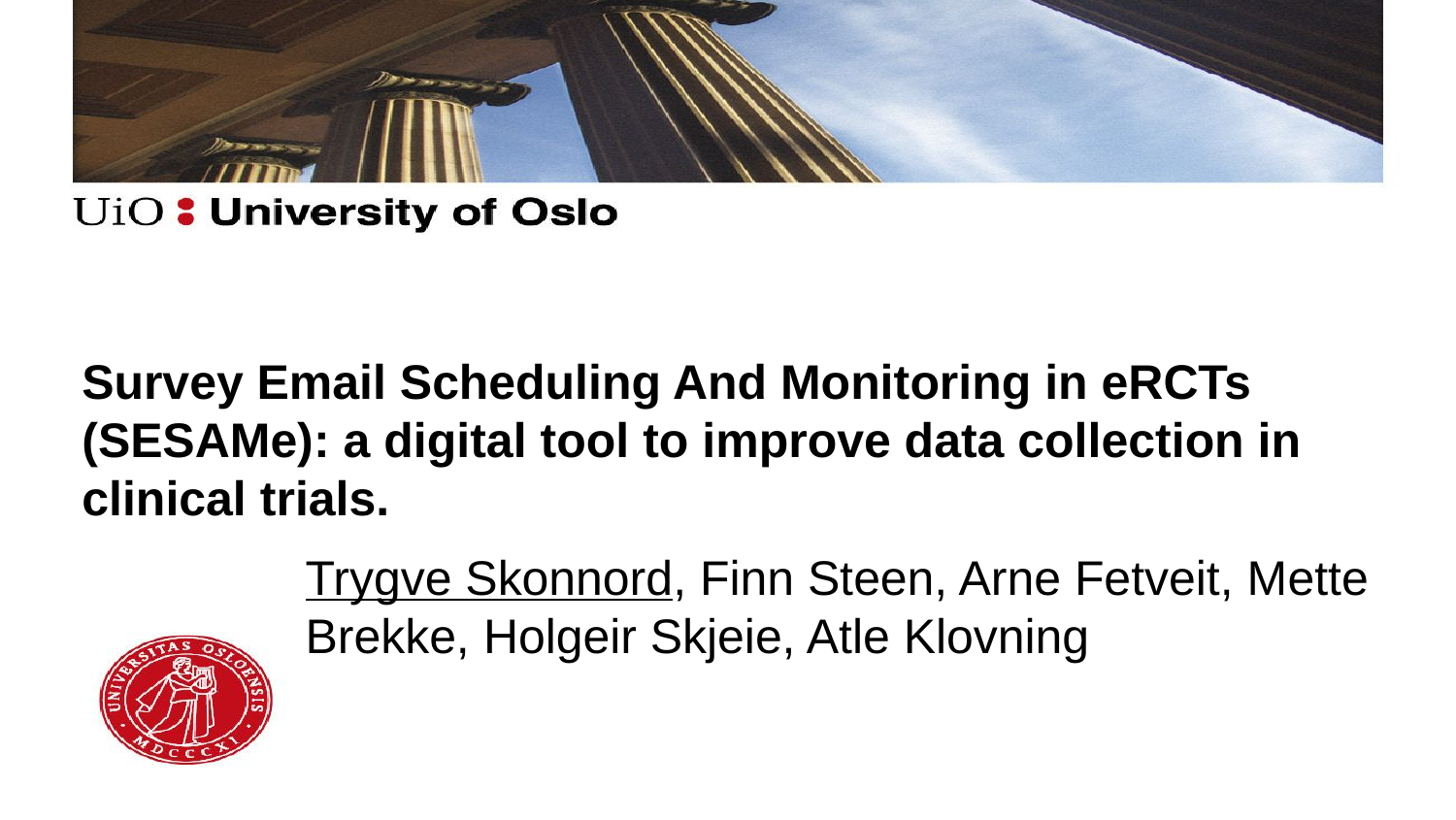

# Survey Email Scheduling And Monitoring in eRCTs (SESAMe): a digital tool to improve data collection in clinical trials.
Trygve Skonnord, Finn Steen, Arne Fetveit, Mette Brekke, Holgeir Skjeie, Atle Klovning

## Slide 2
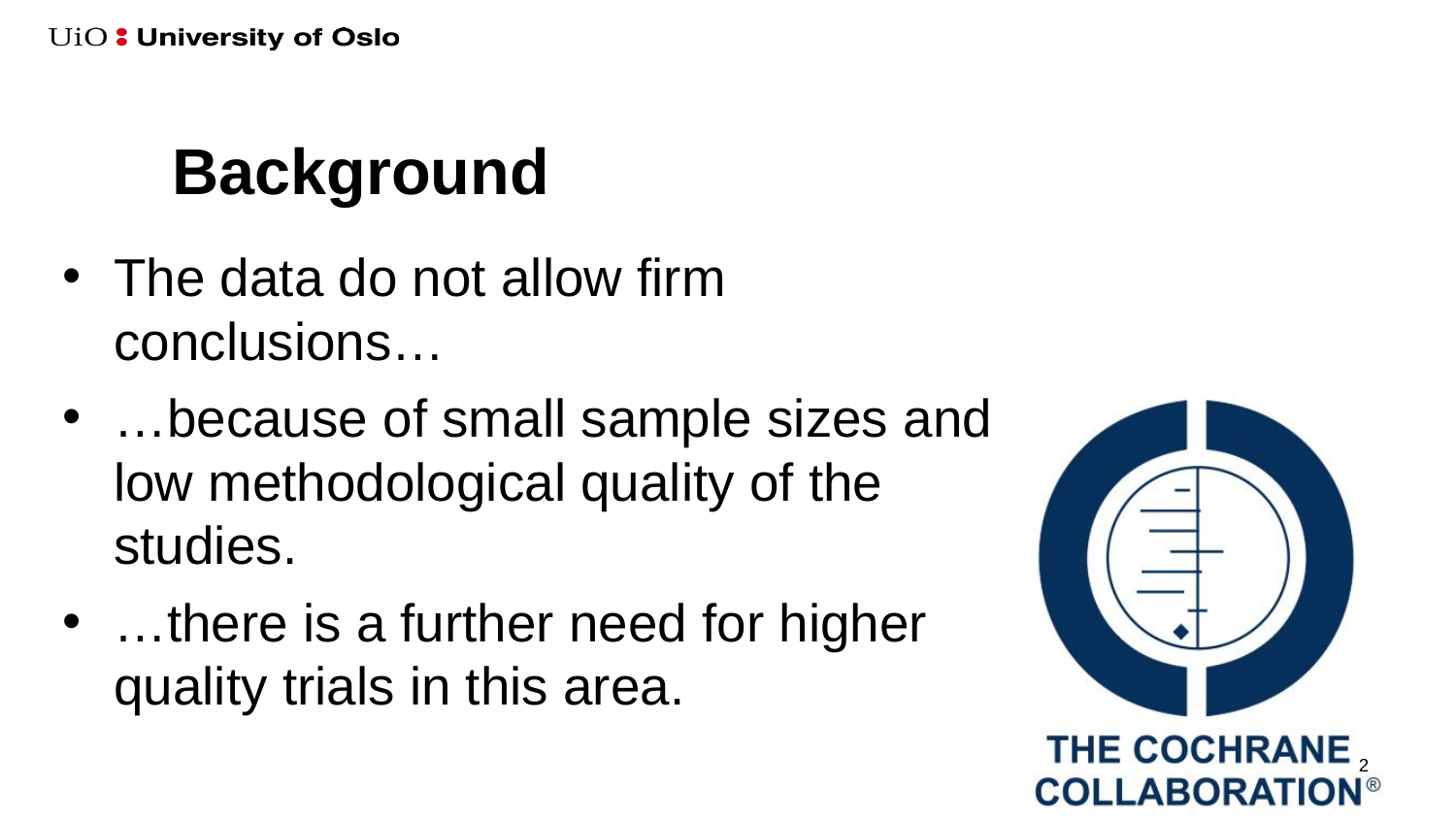

# Background
The data do not allow firm conclusions…
…because of small sample sizes and low methodological quality of the studies.
…there is a further need for higher quality trials in this area.
<number>

## Slide 3
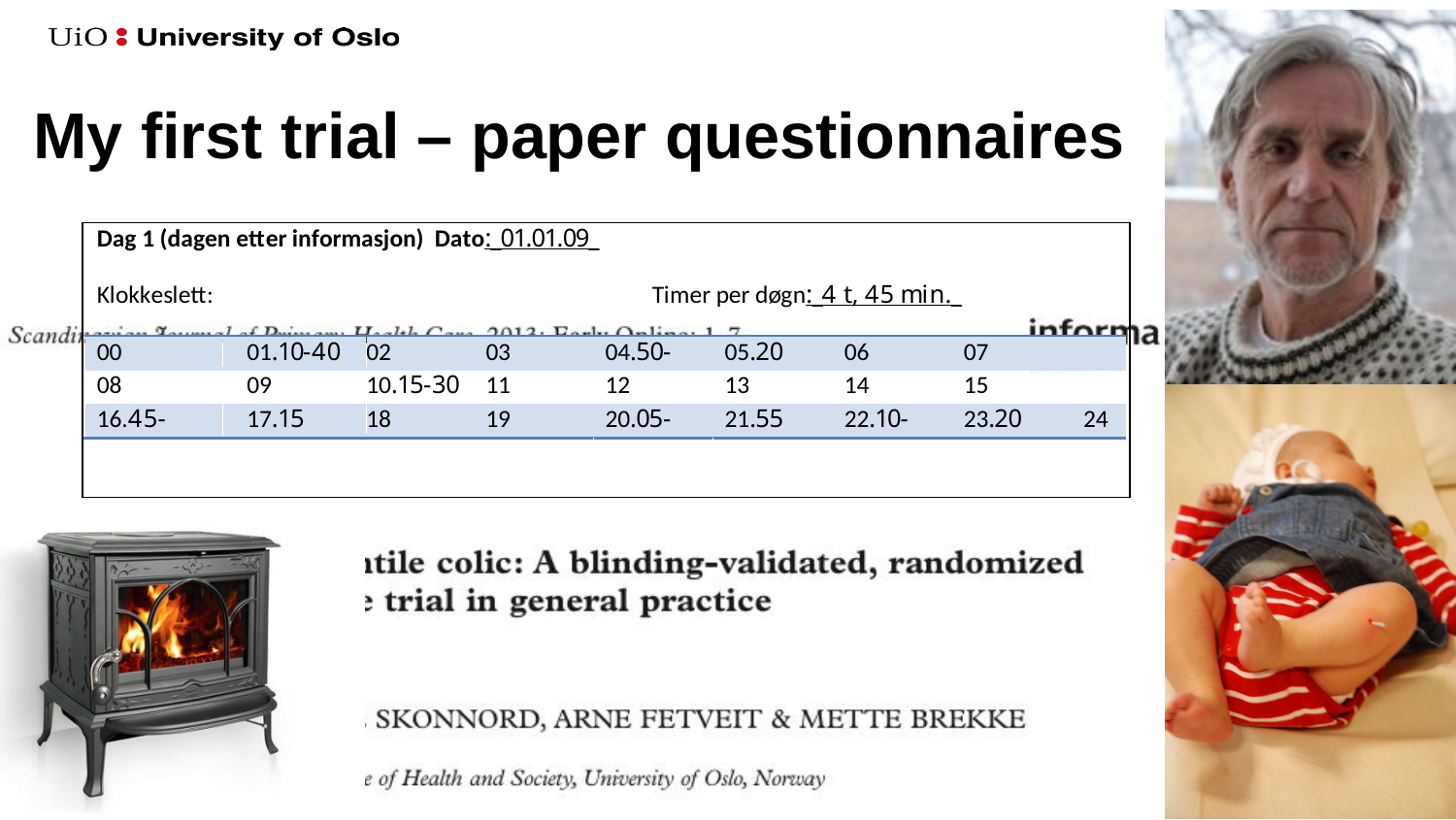

# My first trial – paper questionnaires
<number>

## Slide 4
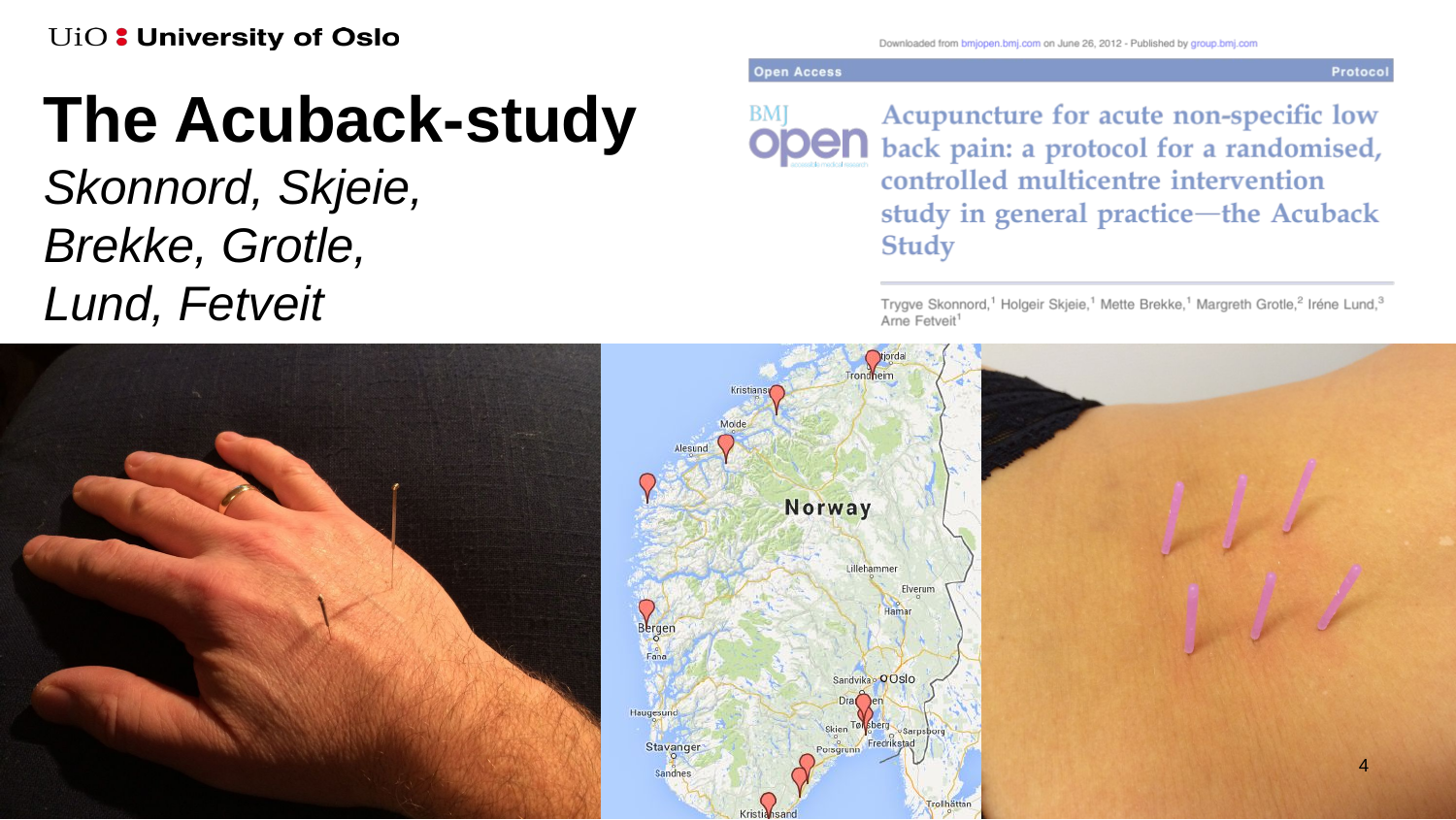

# The Acuback-study Skonnord, Skjeie, Brekke, Grotle, Lund, Fetveit
<number>

## Slide 5
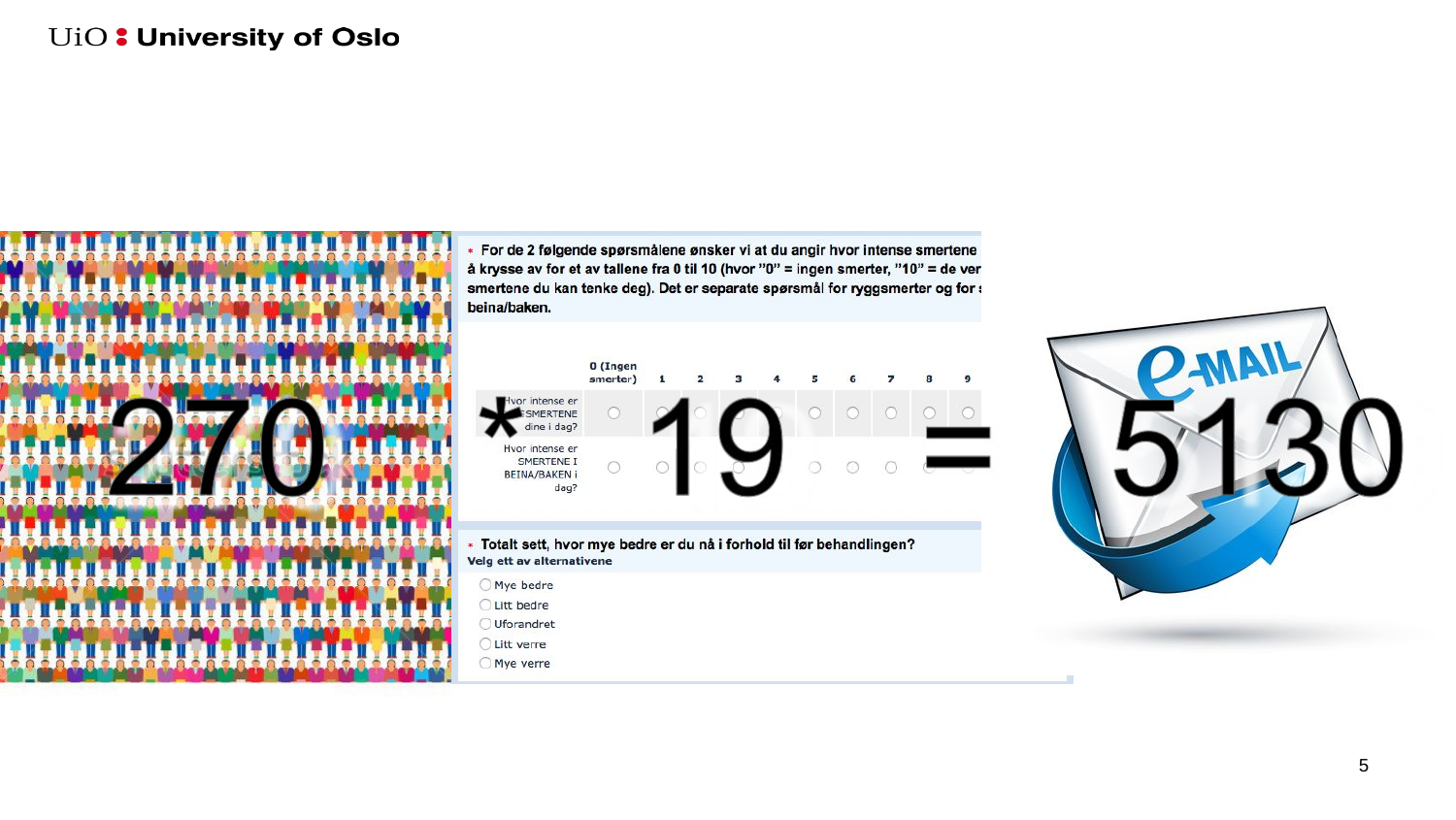

<number>

## Slide 6
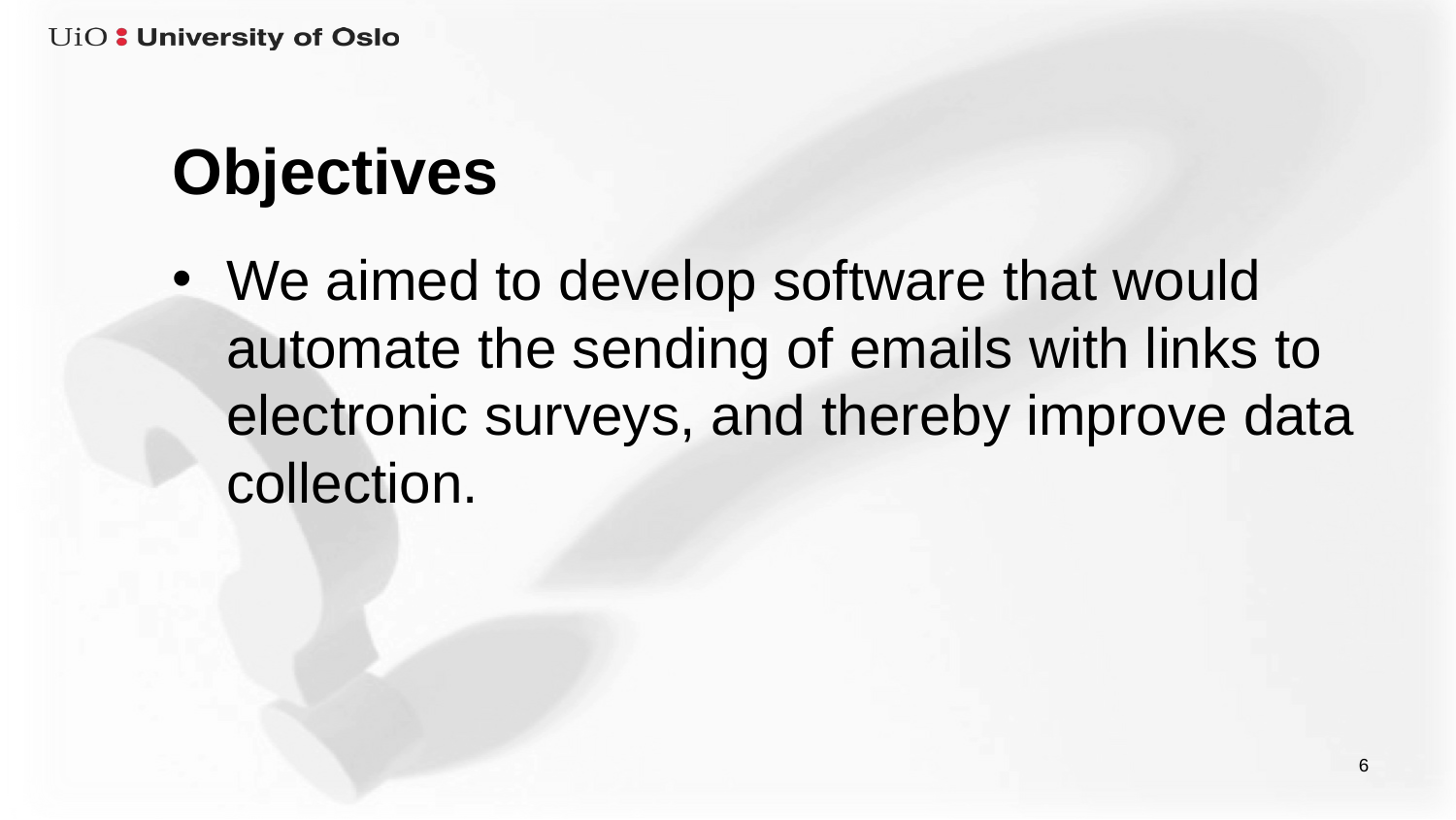

# Objectives
We aimed to develop software that would automate the sending of emails with links to electronic surveys, and thereby improve data collection.
<number>

## Slide 7
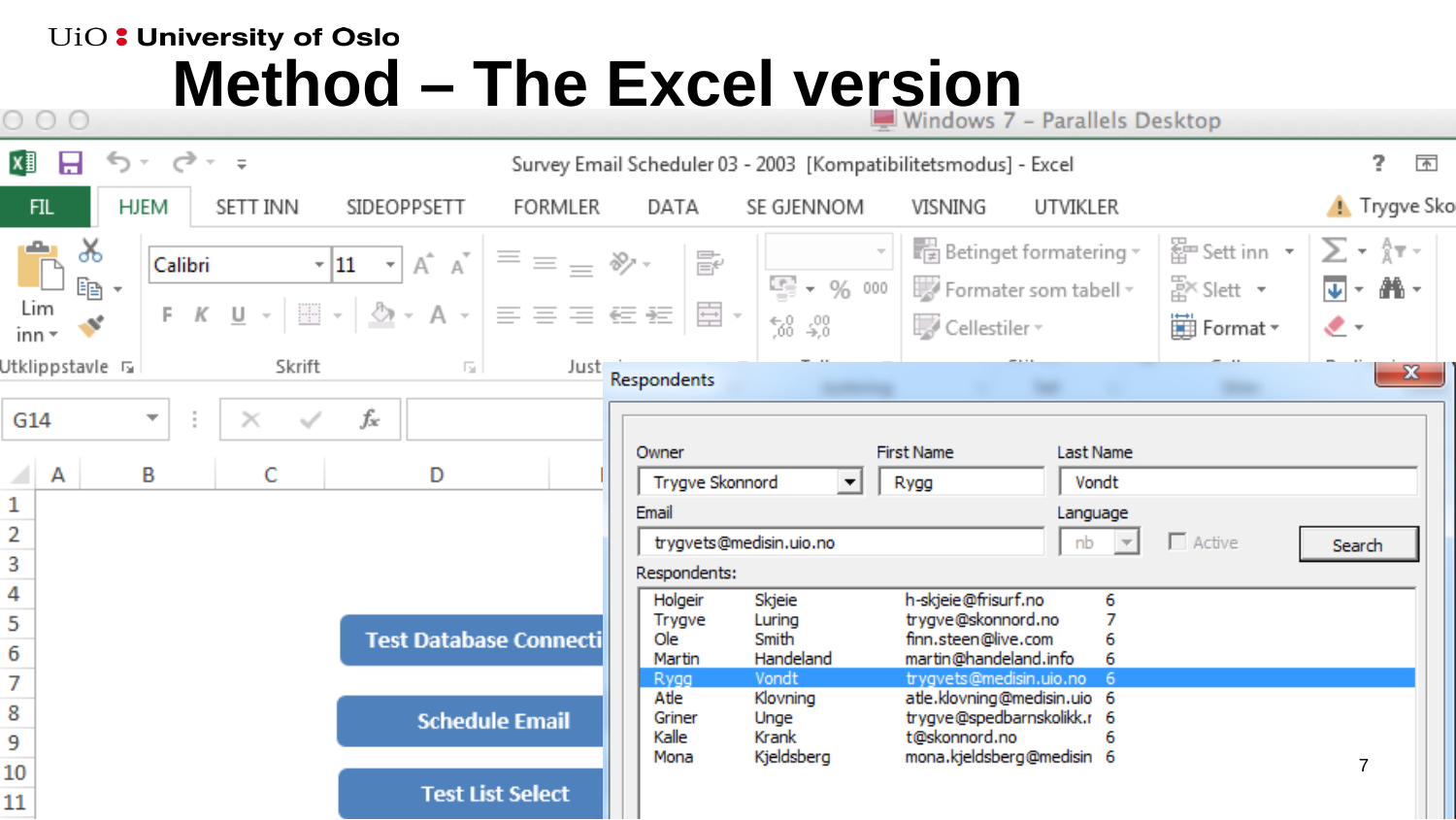

# Method – The Excel version
<number>

## Slide 8
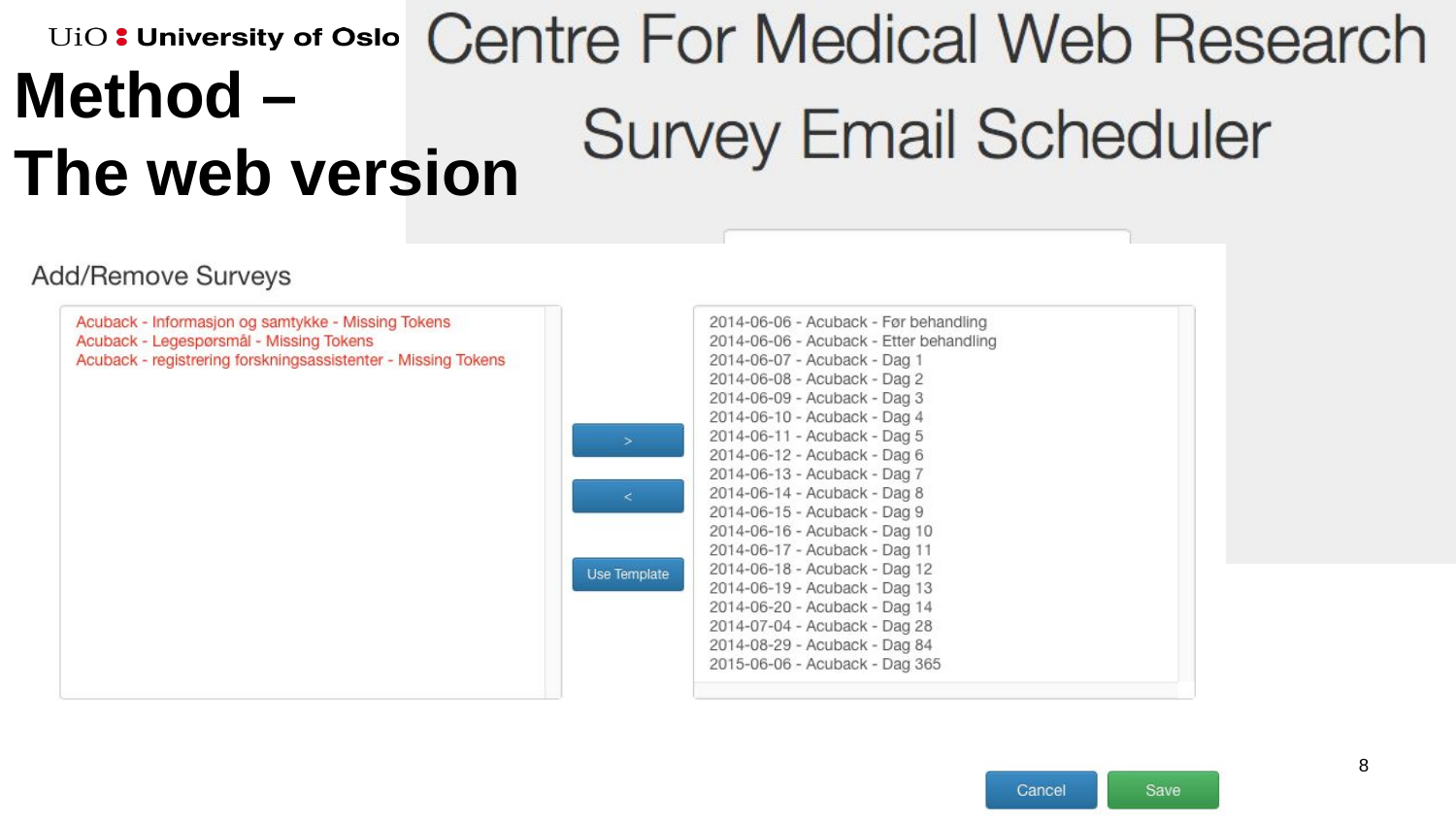

# Method – The web version
<number>

## Slide 9
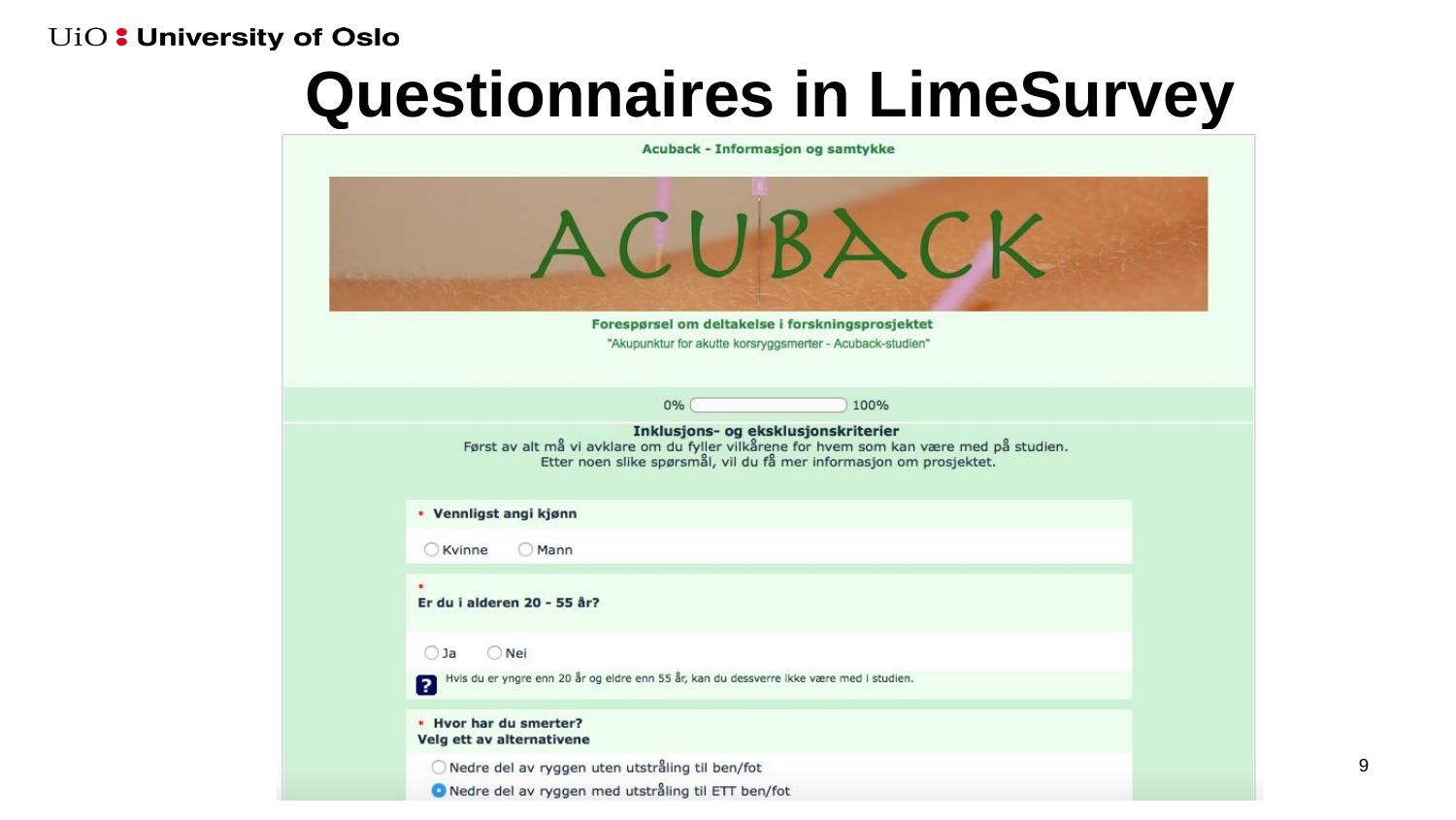

# Questionnaires in LimeSurvey
<number>

## Slide 10
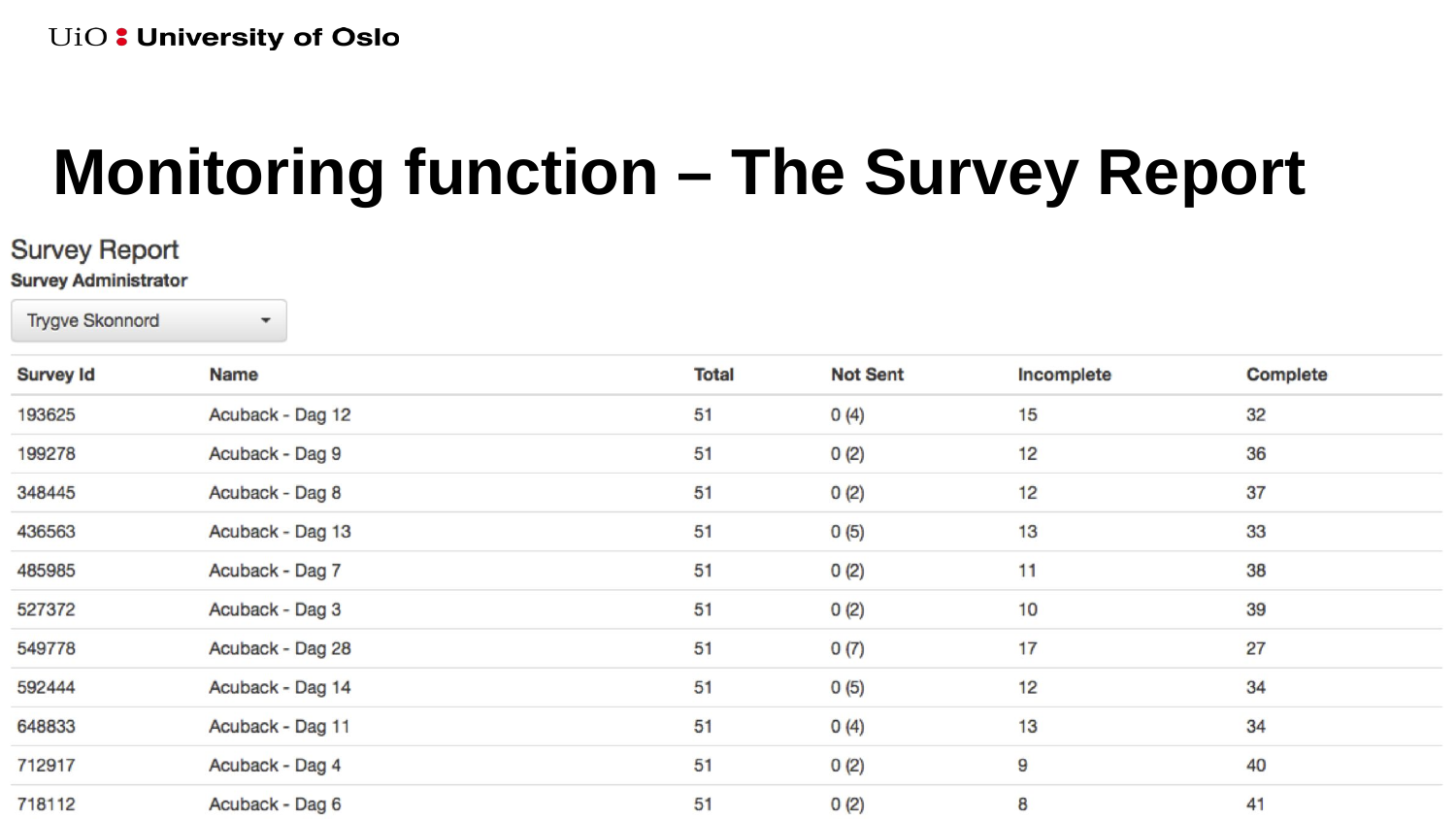

# Monitoring function – The Survey Report
<number>

## Slide 11
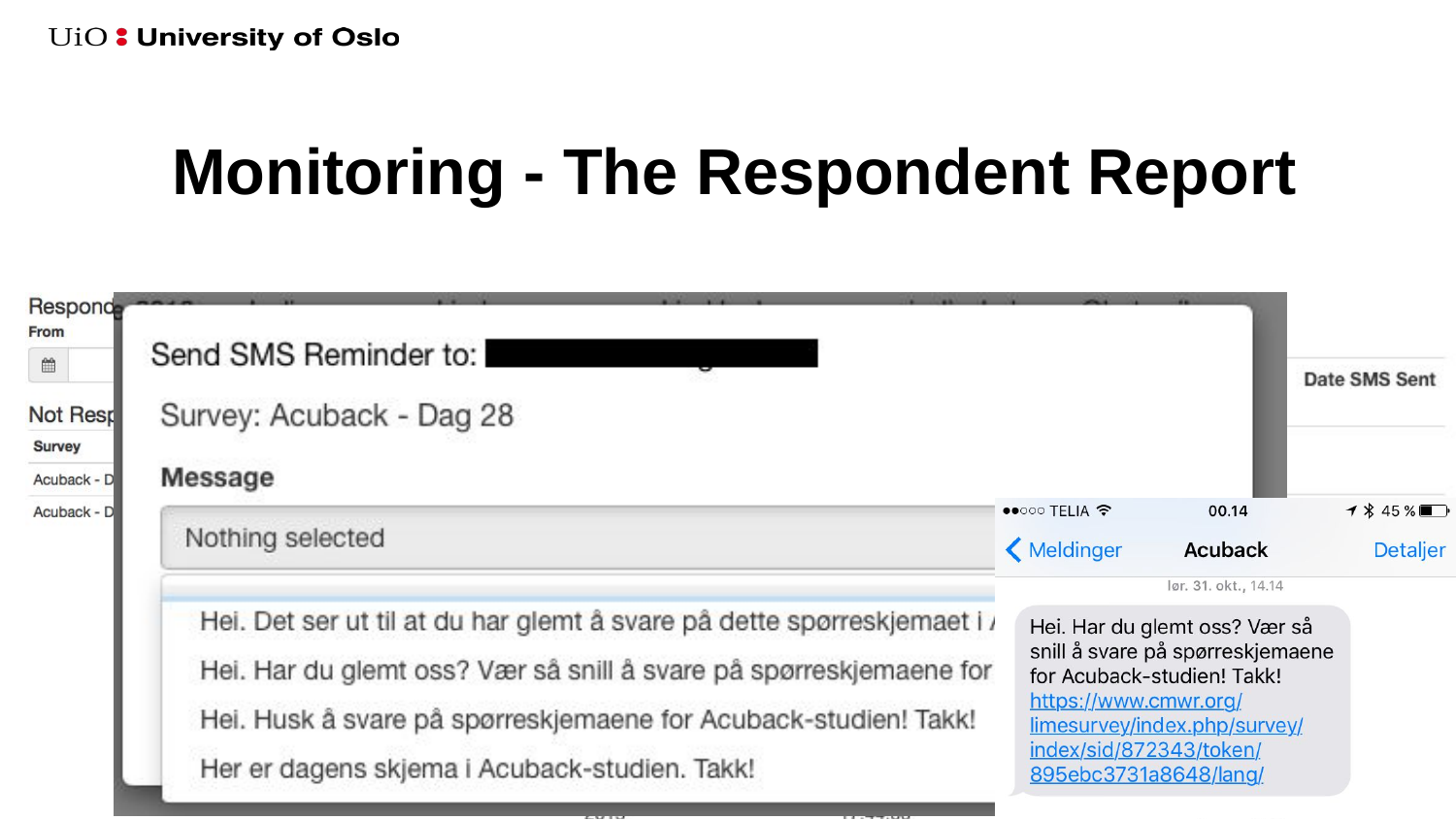

# Monitoring - The Respondent Report
<number>

## Slide 12
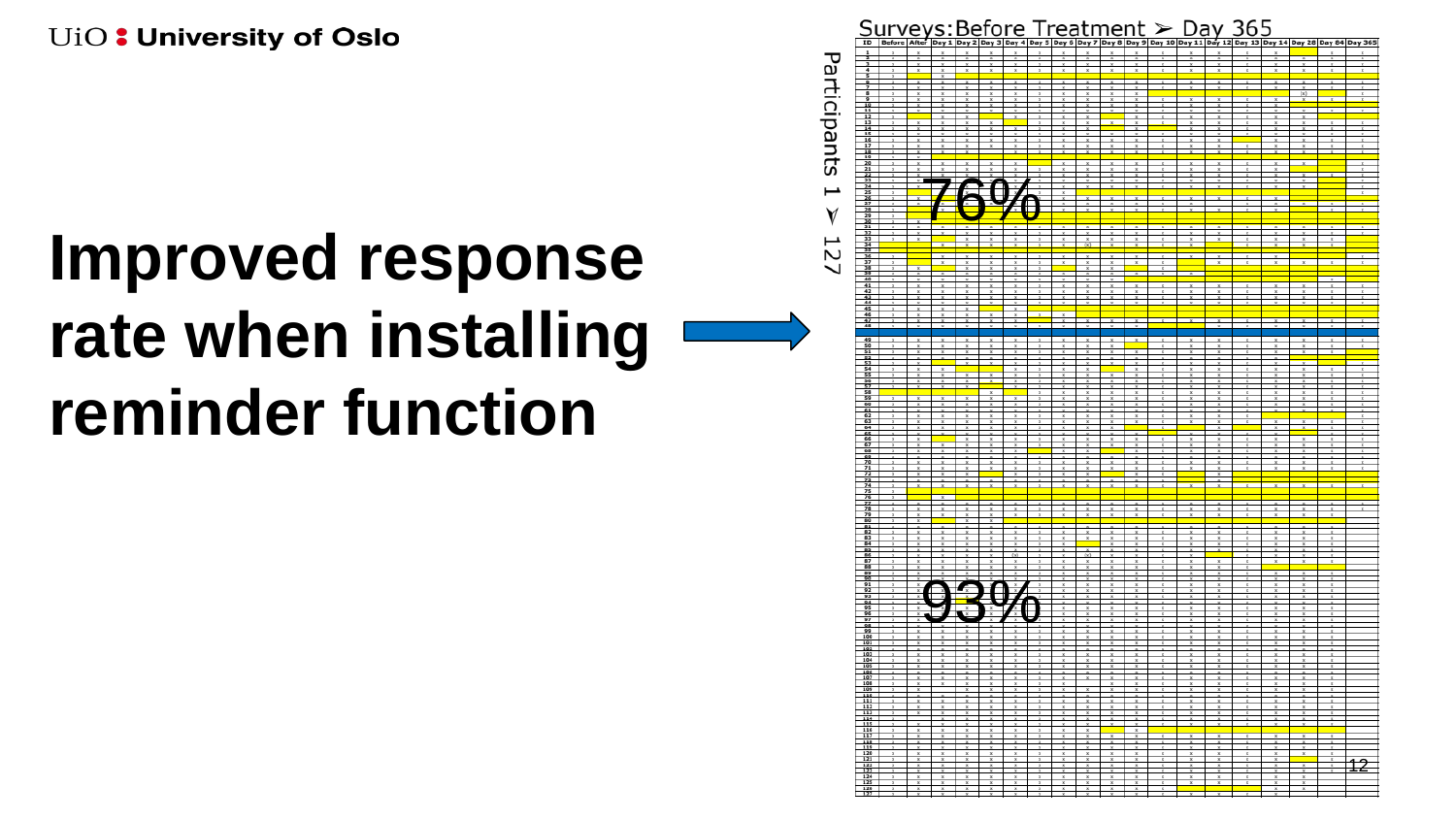

76%
# Improved response rate when installing reminder function
93%
<number>

## Slide 13
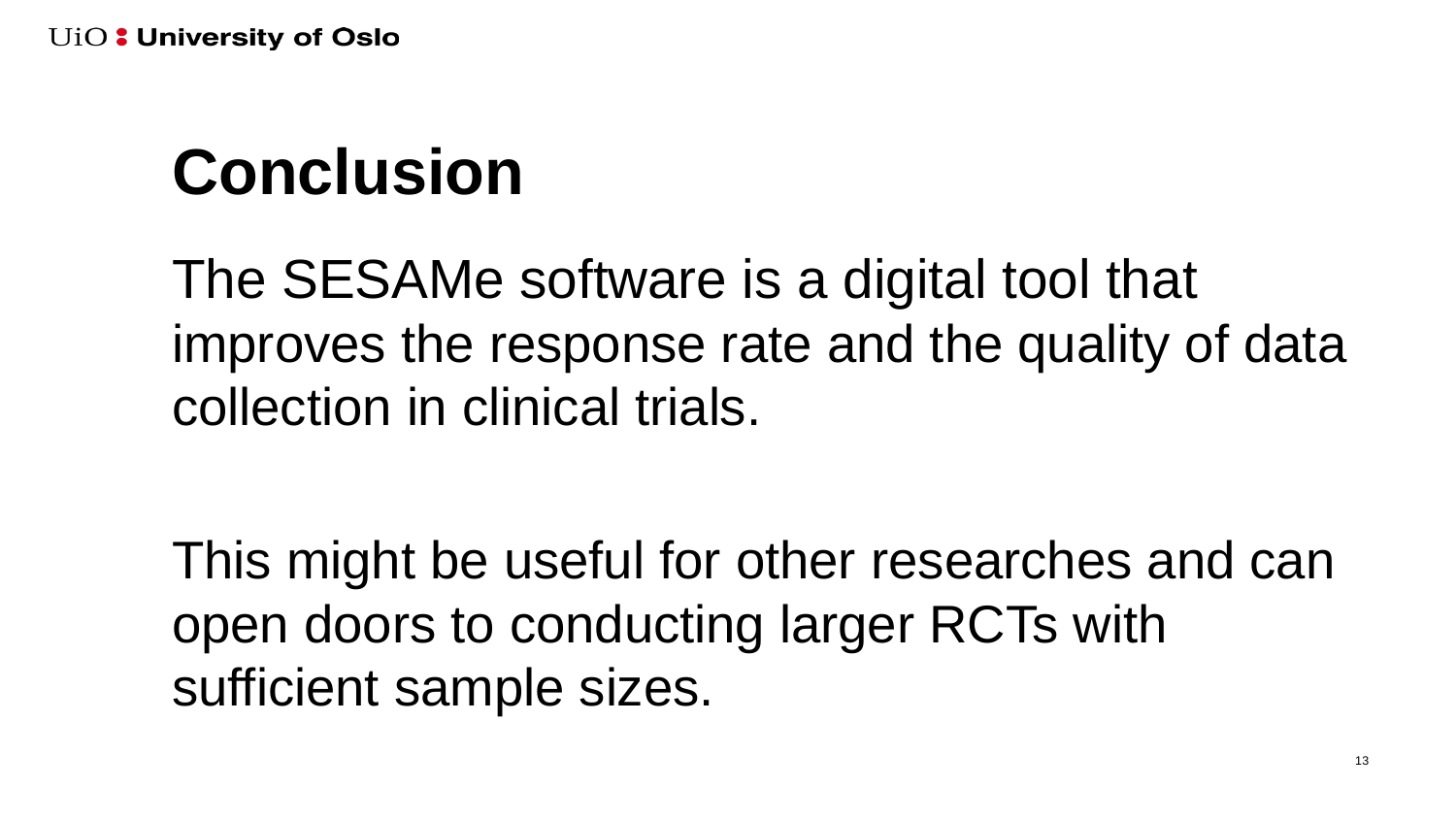

# Conclusion
The SESAMe software is a digital tool that improves the response rate and the quality of data collection in clinical trials.
This might be useful for other researches and can open doors to conducting larger RCTs with sufficient sample sizes.
<number>

## Slide 14
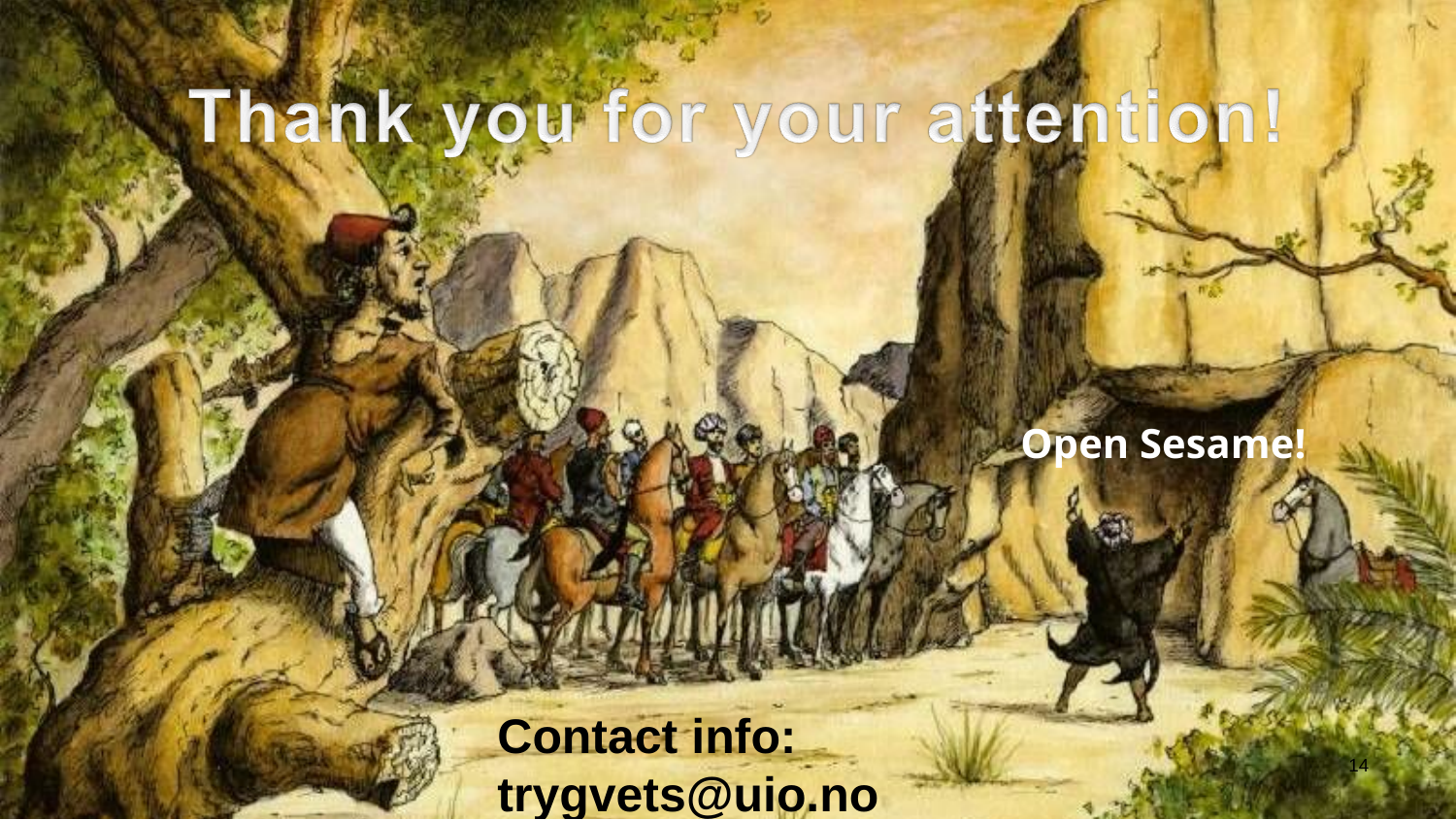

#
Open Sesame!
Contact info:
trygvets@uio.no
<number>
